# Carboxypeptidase M deficiency aggravates complement-induced lung injury

**DOI:** 10.1016/j.isci.2026.117463

**Published:** 2026-09-05

**Authors:** Fatimunnisa Qadri, Chubin Zhang, Anja Schütz, Elena Popova, Beatriz Atocha Czaplinska, Karina Zemechmann, Ramin Greckl, Natalia Alenina, Joao B. Pesquero, André F. Rodrigues, Michael Bader

**Affiliations:** 1 Max Delbrück Center for Molecular Medicine in the Helmholtz Association (MDC), Berlin, Germany; 2 Charité University Medicine Berlin, Berlin, Germany; 3 Health and Medical University, Institute of Molecular Medicine, Potsdam, Germany; 4 Free University Berlin, Institute of Chemistry and Biochemistry, Berlin, Germany; 5 German Center for Cardiovascular Research (DZHK), partner site Berlin, Berlin, Germany; 6 Federal University of Sao Paulo, Department of Biophysics, Sao Paulo, Brazil; 7 University of Lübeck, Institute for Biology, Lübeck, Germany

**Keywords:** anaphylatoxins, carboxypeptidases, acute lung injury, rat model

## Abstract

Carboxypeptidase M (CPM) metabolizes several bioactive peptides by removing their C-terminal arginine residues. It was postulated that the substrates include the anaphylatoxins C3a and C5a generating their less active desArg forms. C3a and C5a are potent mediators of inflammation, shaping both innate and adaptive immune responses. Considering that CPM is a membrane-bound enzyme expressed in multiple organs, we hypothesized that it may protect tissues from anaphylatoxin-driven damage. Using mass spectrometry, we confirmed that CPM efficiently converts C3a and C5a into their desArg forms. We then generated CPM-deficient rats and subjected them to a complement-dependent model of acute lung injury. Compared to controls, CPM-deficient rats exhibited consistently exacerbated lung damage, including higher histological injury scores, increased neutrophil and macrophage infiltration, and elevated expression of inflammatory marker genes. These findings indicate that CPM protects the lung from complement-mediated injury and highlight it as a potential therapeutic target in inflammatory diseases.

## Highlights

• CPM efficiently converts C3a and C5a into their less active desArg forms
• CPM deficiency exacerbates complement-dependent acute lung injury in rats
• CPM loss increases immune cell infiltration and inflammatory gene expression
• CPM protects lung tissue from anaphylatoxin-driven inflammatory damage

## Graphical abstract

**Figure:**
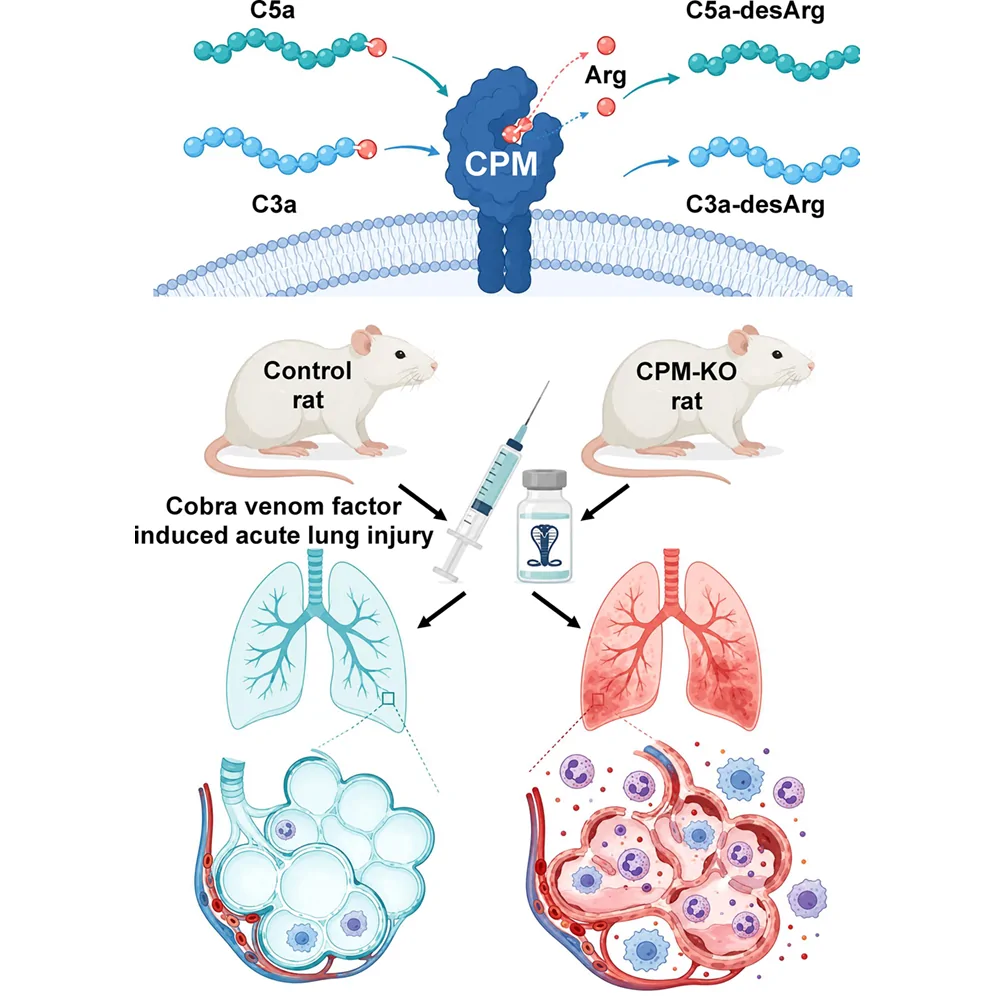

## Teaser

Biochemistry; Molecular biology; Immunology

## Introduction

The complement system is a central component of innate immunity and a critical bridge to adaptive immune responses.^1^^,^^2^ Comprising more than 30 soluble and membrane-bound proteins, the complement cascade is activated through three main pathways, the classical, lectin, and alternative pathways, all converging on C3 cleavage and subsequent activation of C5. While essential for host defense against pathogens, dysregulated or excessive complement activation is increasingly recognized as a major driver of inflammation and tissue damage in a wide range of diseases, including COVID-19.^2^^,^^3^

Among the biologically most potent complement effector molecules are the anaphylatoxins C3a and C5a, which are generated during complement activation by proteolytic cleavage of C3 and C5, respectively.^4^ These small proteins exert strong immunomodulatory effects through their receptors, C3aR, C5aR1 (CD88), and C5aR2 (C5L2). C3a and C5a regulate leukocyte recruitment, vascular permeability, cytokine production, and cell survival, thereby shaping both innate and adaptive immune responses.

C5a is generally considered the most potent anaphylatoxin and is a key mediator of inflammatory pathology.^5^ It acts as a strong chemoattractant for neutrophils and monocytes, primes myeloid cells for enhanced reactive oxygen species production, and promotes the release of pro-inflammatory cytokines. Excessive C5a signaling has been implicated in the pathogenesis of sepsis, autoimmune diseases, ischemia-reperfusion injury, and chronic inflammatory disorders.

C3a, once regarded primarily as a weaker inflammatory mediator, is now recognized as a multifunctional regulator of immunity.^2^^,^^6^ Beyond its roles in chemotaxis and vascular activation, C3a signaling influences T cell polarization, dendritic cell function, and tissue regeneration. Aberrant C3a-C3aR signaling has been linked to allergic diseases, neuroinflammation, metabolic disorders, and cancer. Importantly, C3a and C5a often act in a coordinated yet non-redundant manner, and the balance between their signaling pathways can critically determine disease outcomes.^2^

C5a and C3a are metabolized by carboxypeptidases N (CPN) and B2 (CPB2 or TAFI) in plasma generating their less active desArg-forms.^7^^,^^8^ C5a-desArg is 10– to 100-fold less active on the C5aR1 receptor and C3a-desArg does not bind at all to the C3aR receptor; however C5a-desArg maintains activity on the C5aR2 receptor.^9^^,^^10^^,^^11^ Membrane-bound carboxypeptidase M (CPM) has also been postulated to catalyze the release of the C-terminal arginine from these anaphylatoxins,^12^ but this was, to the best of our knowledge, never experimentally shown using full-length C5a and C3a. CPM is expressed predominantly in lung, bone marrow, placenta, and kidney, and plays a central role in multiple peptide systems by, among others, converting bradykinin to desArg9 bradykinin and metabolizing CXCL12.^12^^,^^13^ Unlike CPN and CPB2, CPM is linked to the plasma membrane of cells by a GPI-anchor^12^^,^^13^ and thereby tissue-resident, making it a strong candidate for local protection against complement-mediated tissue damage. To test this hypothesis, we demonstrated that CPM converts C5a and C3a to their desArg forms and then generated CPM-deficient rats and subjected them to cobra venom factor (CVF)-induced lung injury. This acute lung disease model is driven by complement activation since CVF is a C3b homolog that forms a C3/C5 convertase and generates C3a and C5a.^14^^,^^15^^,^^16^^,^^17^

## Results

### CPM cleaves the C-terminal arginine from C3a and C5a

**Figure 1:**
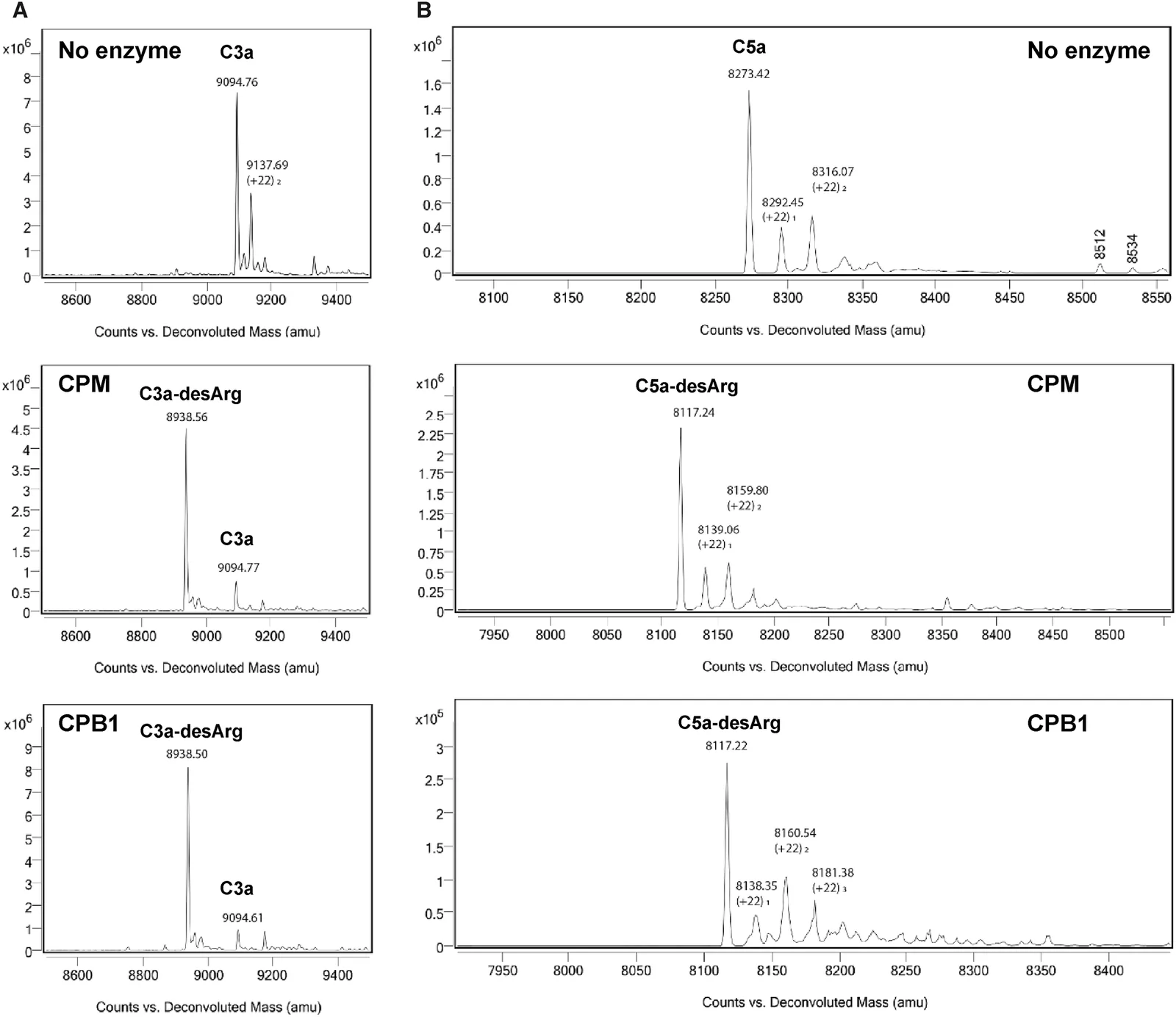
Intact mass analysis of C-terminal arginine removal *in vitro* *In vitro* carboxypeptidase assays of complement component C3a (A) and C5a (B) were analyzed by intact mass spectrometry to monitor C-terminal Arg removal (−156.19 Da) after 5-min incubation in the absence of enzyme (top, control) or in the presence of carboxypeptidase M (CPM, middle) or carboxypeptidase B1 (CPB1, bottom). The calculated masses are 9,094.65 Da for C3a (C3a-desArg 8,938.46 Da) and 8,273.63 Da for C5a protein (C5a-desArg 8,117.44 Da). Signals annotated as (+22)_n_ denote sodium adduct species arising from replacement of protons by Na^+^, corresponding to a mass increase of +22 Da per adduct.

In order to verify that CPM can metabolize C5a and C3a into their desArg forms, we incubated recombinant CPM with recombinant C5a and C3a and measured the mass of the peptides after 5 min incubation at 37°C. C5a and C3a were completely converted to their desArg forms (Figure 1) by CPM. Recombinant CPB1 served as positive control and also catalyzed the complete removal of the C-terminal arginine from both anaphylatoxins.

### Genetic ablation of CPM abolishes carboxypeptidase activity in the lung

**Figure 2:**
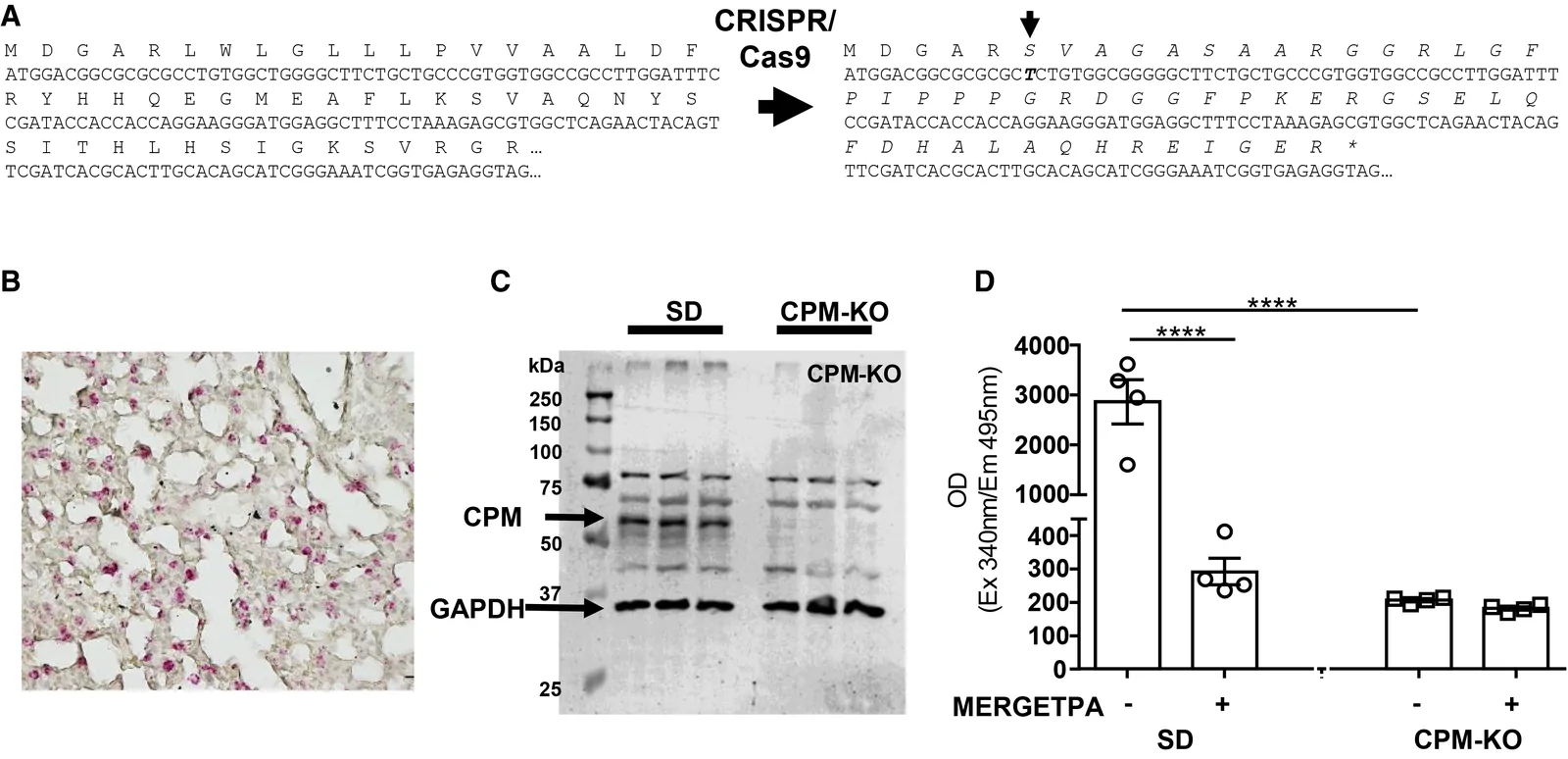
Generation of CPM-knockout rats (A) CPM-deficient rats were generated using CRISPR-Cas9 technology which induced a frameshift mutation in the *Cpm* gene (introduction of a T, indicated by an arrow). (B) Normal expression of CPM-mRNA (red dots) in the lung tissue detected by RNAScope. The genetic alteration resulted in a complete loss of CPM protein, as confirmed by western blot analysis (C, *n* = 3 rats/group) and a strong reduction of carboxypeptidase activity (D, *n* = 4 rats/group) in lung tissue. CPM, carboxypeptidase M, GAPDH, glyceraldehyde-3-phosphate dehydrogenase, SD, Sprague Dawley control rats, CPM-KO, CPM-knockout rat, MERGETPA, DL-2-mercaptomethyl-3-guanidinoethylthiopropanoic acid. ∗∗∗∗, *p* < 0.0001.

Since CPM is expressed on the surface of cells, we hypothesized that it could locally protect tissues from C5a and C3a induced damage. Therefore, we generated CPM-knockout (KO) rats using CRISPR-Cas9 technology by introducing a T into the 5′-end of the coding sequence leading to a frameshift and early termination of the protein (Figure 2A). In the lung, CPM is normally expressed in epithelial cells of the bronchi (Figure 2B). Western blot and enzyme activity assays confirmed the absence of the enzyme in the lung of CPM-KO rats (Figures 2C and 2D, respectively). MERGETPA, a selective inhibitor of CPN, CPB1, CPB2, and CPM,^18^ reduced the carboxypeptidase activity to the same extent as CPM-ablation with only a minor additional decrease observed in CPM-KO samples. Moreover, kidney and bone marrow showed much lower CPM activity, which was also markedly reduced in CPM-KO rats (Figures S1A and S1B, respectively). These findings indicate that CPM represents the predominating carboxypeptidase activity in these tissues. The residual activity observed in these tissues is probably due to other carboxypeptidases metabolizing the substrate and not being inhibited by MERGETPA. We detected low expression of CPN1 and CPB2 lungs, which remained unaltered in CPM-KO rats (Figures S1C and S1D). In liver, the expression of CPN1 did also not change (Figure S1E), but CPB2 expression was significantly reduced (Figure S1F). The reason for this regulation remains enigmatic.

### Loss of CPM aggravates complement-induced acute lung injury

**Figure 3:**
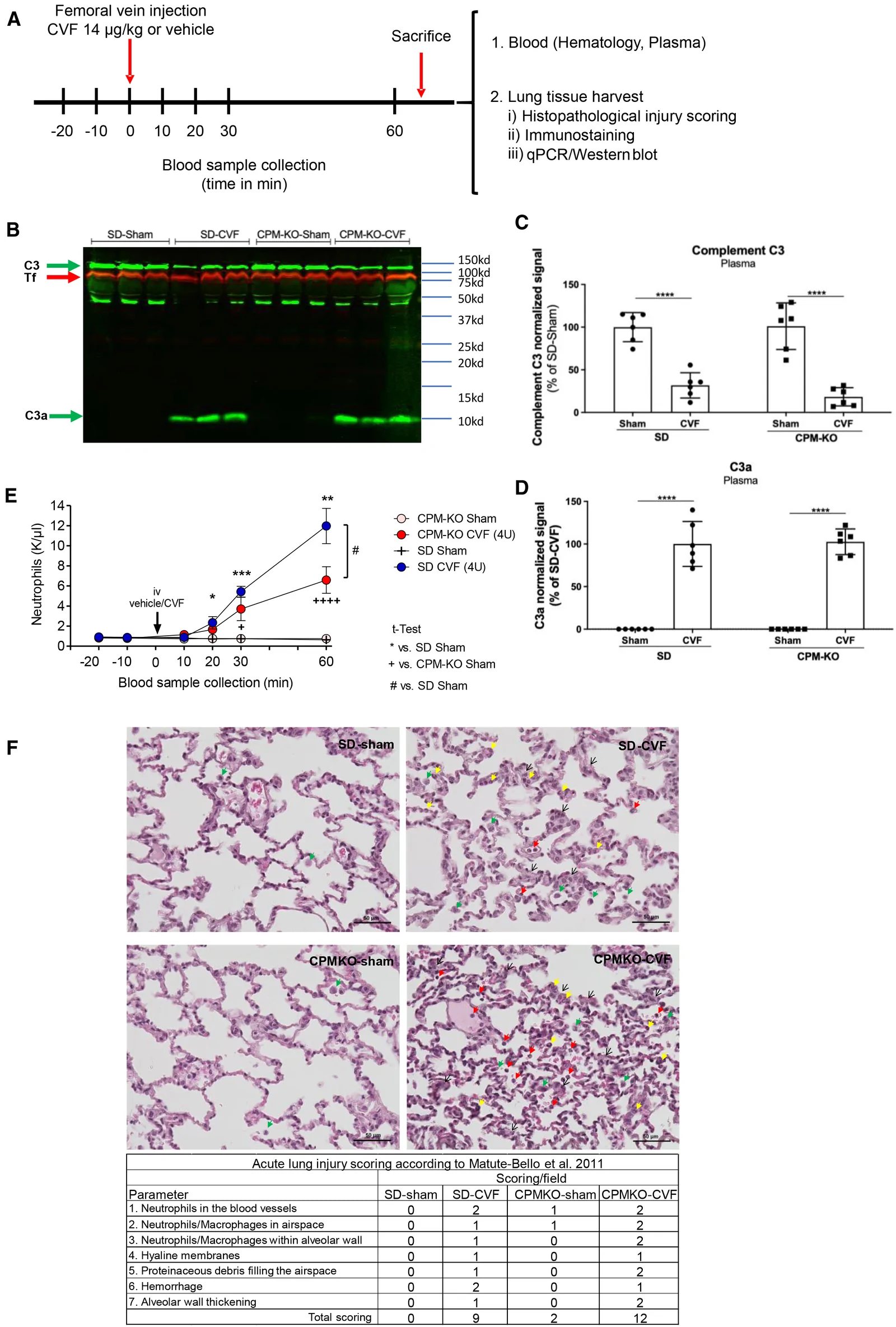
Induction of acute lung injury (A) Schematic overview of the experimental setup. Acute lung injury was induced by intravenous injection of 4 IU CVF in male SD (SD-CVF, *n* = 6) and CPM-KO (CPM-KO CVF, *n* = 6) rats. Vehicle-injected rats were used as controls (SD-sham, *n* = 6, CPM-KO sham, *n* = 6 rats). Blood samples were collected at the indicated time points from the femoral vein before and after injection. Animals were sacrificed 60 min after CVF administration and lung tissue was harvested for further analysis. (B–D) Western blot analysis showing the CVF-induced cleavage of C3 to C3a in plasma 60 min after injection with corresponding quantitative analysis. (E) Blood neutrophil counts (Neutro). (F) Representative lung histology used for injury scoring according to Matute-Bello et al.^19^ with modifications. C3, complement factor 3; C3a, complement factor C3a (including C3a-desArg); red arrow, neutrophils in alveolar space; green arrow, macrophages in alveolar space; yellow arrow, neutrophils in interstitial space; black arrow, alveolar wall thickening. ∗∗∗∗, *p* < 0.0001.

CPM-KO and wild-type (WT) Spraque-Dawley (SD) rats were subjected to the model of CVF induced lung injury, which is known to be mainly driven by the complement anaphylatoxins, C5a and C3a^14^ (Figure 3A). Intravenous *bolus* injection of 4 IU/kg recombinant CVF derived from *Naja kaouthia* resulted in rapid complement activation in both WT and CPM-KO rats, as demonstrated by western blot analysis showing efficient cleavage of C3 and generation of C3a or C3a-desArg (Figures 3B and 3D). Of note, these two forms could not be distinguished in this assay due to the small difference in mass and the lack of a specific antibody.

**Figure 4:**
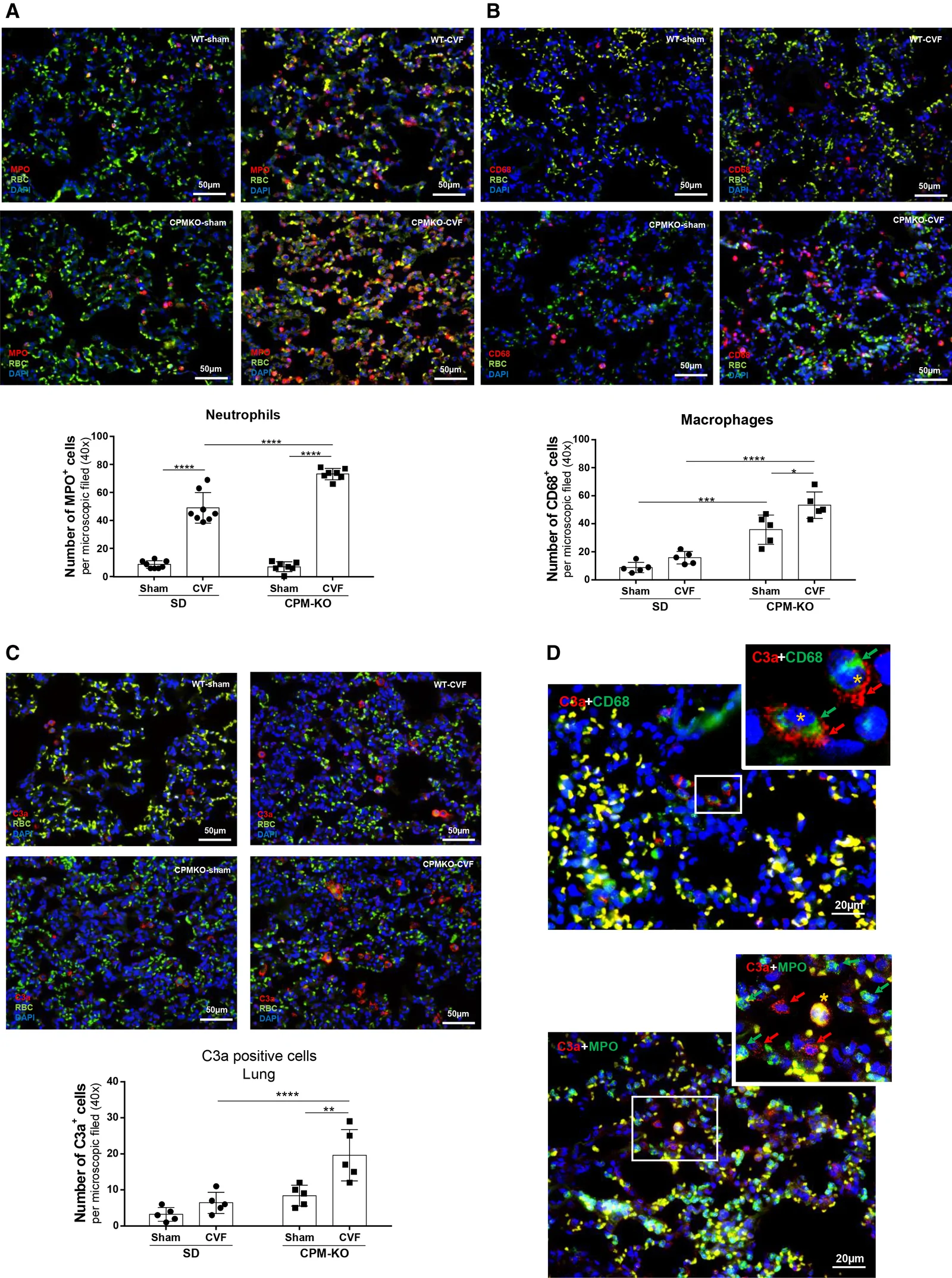
Pulmonary cell infiltration after acute lung injury Immunohistochemistry using antibodies against (A) MPO and (B) CD68 revealed increased infiltration of neutrophils (*n* = 7 rats of each group) and macrophages (*n* = 5 rats of each group), respectively, in the lung of injured rats (SD-CVF and CPMKO-CVF), compared to controls (SD-sham, CPMKO-sham), representative images (top) and quantitative analysis (bottom). (C) A neoepitope-specific antibody against human C3a detected C3a-positive cells in the lungs of injured rats (*n* = 5 rats of each group). (D) Double labeling for C3a and CD68 showed mainly double labeled macrophages (top). Double labeling for C3a (red arrows) and myeloperoxidase (MPO, green arrows) mostly C3a-negative neutrophils, yellow asterisks show few double-labeled cells (bottom); two different magnifications are shown. ∗, *p* < 0.05, ∗∗∗, *p* < 0.001, ∗∗∗∗, *p* < 0.0001.

Hematological analysis revealed a robust and progressive increase in circulating neutrophils number starting 20 min after CVF treatment. This response was markedly blunted in the CPM-KO rats probably due to increased infiltration of these cells into the lung (Figure 3E). Hematoxylin and eosin-stained lung sections revealed clear morphological features of acute lung injury following CVF treatment, including inflammatory cell infiltration, alveolar wall thickening, and accumulation of cells within the alveolar space (Figure 3F). These changes were markedly more pronounced in CPM-KO rats, which exhibited dense infiltration of neutrophils and macrophages, as well as more severe disruption of alveolar architecture. Semi-quantitative scoring according to Matute-Bello et al.^19^ confirmed successful induction of acute lung injury, which was even more pronounced in CPM-KO rats than in SD controls (Figure 3F). Accordingly, neutrophils had massively invaded the lung after CVF treatment, as detected by immunostaining for MPO, and this accumulation was more pronounced in CPM-KO rats (Figure 4A). In contrast, macrophages were already more abundant in CPM-KO lungs under baseline conditions and showed only a modest increase one hour after CVF treatment, suggesting that macrophage recruitment is initiated but not yet fully developed at this stage. This increase reached statistical significance only in CPM-KO rats (Figure 4B). C3a positive cells were detected by immunofluorescence staining with a specific antibody recognizing a neoepitope exposed after the cleavage of the precursor proteins and, thus, not labeling full-length C3. Their number increased significantly only in CPM-KO rats after CVF treatment (Figure 4C). Colocalization analyses showed that mainly macrophages and, to a lesser extent, neutrophils were stained by C3a on the cell surface (Figure 4D). It is of note that C3a and C3a-desArg are indistinguishable in these assays, due to the lack of specific antibodies. However, given that only C3a can bind to C3aR, whereas C3a-desArg exhibits markedly lower affinity for this receptor, the detected signal likely reflects biologically active C3a bound to the cell surface.

**Figure 5:**
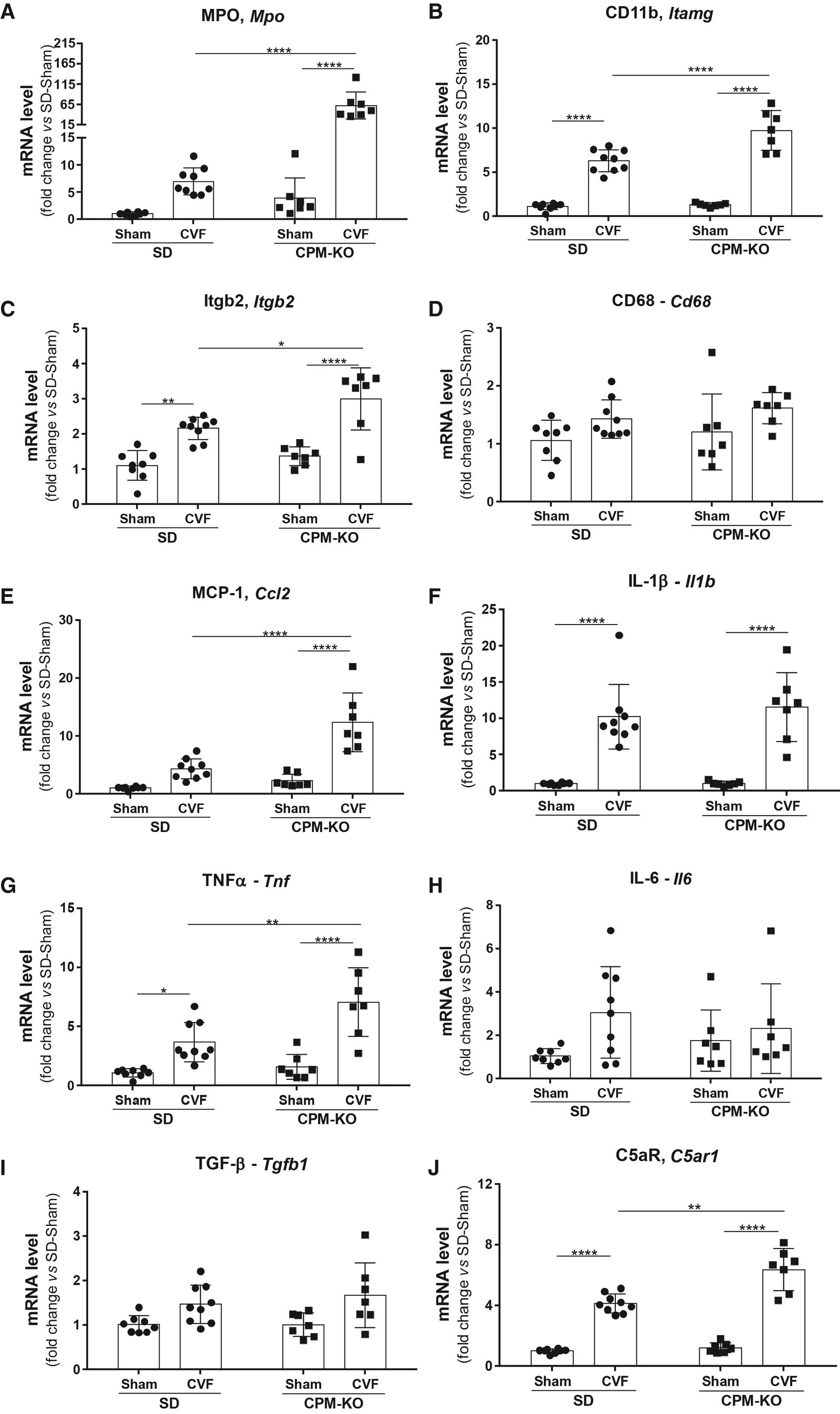
Gene expression in injured lungs Gene expression was quantified in lung tissue of injured (SD-CVF, *n* = 5 rats, CPM-KO-CVF, *n* = 5 rats) and control rats (SD-Sham, *n* = 5 rats CPM-KO-Sham, *n* = 5 rats) by RT-qPCR using gene-specific primers for (A) myeloperoxidase (MPO), (B) CD11b, (C) integrin subunit β2 (Itgb2), (D) CD68, (E) monocyte-chemoattractant protein 1 MCP-1), (F) interleukin 1β (IL1β), (G) tumor-necrosis factor α (TNFα), (H) interleukin 6 (IL6), (I) transforming growth factor β (TGF-β), and (J) complement factor C5a receptor 1 (C5aR1). ∗, *p* < 0.05, ∗∗, *p* < 0.01, ∗∗∗∗, *p* < 0.0001.

Gene expression analysis corroborated the histological data showing increased expression of the neutrophil markers MPO, CD11b, and Itgb2 in CVF treated rats, with a more pronounced upregulation in CPM-KO animals (Figures 5A–5C). Expression of CD68, a marker for macrophages, did not differ between the groups (Figure 5D). Furthermore, the expression of other inflammation markers, such as MCP-1, IL1β, and TNFα, but not IL6 and TGFβ, was markedly elevated in CVF-treated rats with MCP-1 and TNFα mRNA levels being significantly higher in CPM-KO animals (Figures 5E–5I). C5aR1 followed a similar pattern of expression showing a significantly greater increase in CPM-KO rats than in SD controls (Figure 5J). Since C5aR1 is highly expressed in neutrophils, these data further support enhanced infiltration of these cells into CPM-deficient lungs after CVF treatment.

## Discussion

In this study we show that CPM effectively inactivates the complement anaphylatoxins, C5a and C3a, by removing their C-terminal arginine. A previous report showed that a short C-terminal fragment (residues 70–77) of C3a is a substrate of CPM,^20^ but to the best of our knowledge, full-length anaphylatoxins have not yet been employed in this context. In contrast, the conversion of C5a and C3a to their desArg forms has been repeatedly reported for CPN, CPB1, and CPB2.^7^^,^^8^^,^^21^

The consequences of CPN or CPB2 deficiency across different disease models leading to a prolonged half-life of anaphylatoxins have previously been described. While CPB2-deficient mice are protected from sepsis-induced mortality, likely due to stabilization of C3a, which is protective in this disease, CPN-deficient animals exhibit increased mortality in shock models due to an overactivity of C5a.^22^^,^^23^^,^^24^ In a model of hemolytic uremic syndrome, both mouse lines showed exacerbated disease, but CPN-deficient mice were less severely affected than animals lacking CPB2. Moreover, the CVF-induced lung injury model was more aggravated in both lines^25^ confirming our data in CPM-KO rats; however, here, CPB2-deficient mice were less affected than CPN-KO animals. In line with these observations, simultaneous pharmacological inhibition of all three carboxypeptidases—CPM, CPN, and CPB2—using MERGETPA results in markedly increased mortality following CVF administration in guinea pigs.^26^

CPM-deficient animals have not yet been described and therefore the relevance of this enzyme in complement dependent diseases remained unclear. To address this, we generated CPM-deficient rats using CRISPR-Cas9 technology introducing a frameshift mutation in the *Cpm* gene that resulted in a complete loss the protein and enzymatic activity. In this newly established rat model, we investigated the protective role of CPM in the lung, since we had shown that it is predominantly expressed in this organ. As we demonstrated that CPM can inactivate the anaphylatoxins, C3a and C5a, we chose the model of acute lung injury induced through CVF-mediated complement activation.

In our study, CPM deficiency aggravated CVF-induced acute lung injury as evidenced by increased infiltration of neutrophils and macrophages along with reinforced expression of inflammatory markers. We suggest that the protective effect of CPM in this disease model is due to its local inactivation of the anaphylatoxins, C3a and C5a, the key mediators of immune cell recruitment. Supporting this hypothesis, we observed elevated levels of C3a on macrophages in CPM-KO rats compared to SD controls following induction of acute lung injury. CPM is expected to convert C3a to C3a-desArg, which has a marked reduced affinity for C3aR. In the absence of CPM, this conversion is impaired resulting in sustained binding of C3a to the cell surface.

Given their central roles in inflammatory and immune-mediated diseases, C3a and C5a have emerged as attractive therapeutic targets.^27^ Complement-directed therapies, particularly those targeting C5 or C5a-C5aR1 signaling, have already demonstrated clinical efficacy, underscoring the translational relevance of understanding anaphylatoxin biology. In light of our findings demonstrating a protective role of CPM in complement-depending acute lung injury we propose that CPM activation may represent an innovative therapeutic strategy for diseases driven by excessive anaphylatoxin activity. Alternatively, administration of recombinant CPM could provide means to directly inactivate C3a and C5a. Such approaches may be particularly effective in inflammatory lung diseases, including acute respiratory distress syndrome or COVID-19,^3^^,^^28^^,^^29^ given the predominant expression of CPM in pulmonary tissue. However, the therapeutic benefit may also extend to other tissues, such as kidney with moderate levels of CPM expression.

### Limitations of the study

A limitation of our study is the lack of direct quantification of C5a and C3a and their respective desArg metabolites in the rat lung tissue, as no validated methods are currently available for this purpose. Commercial antibodies and ELISA kits for rats do not discriminate between C5a and C5 or between C3a and C3 since the relevant epitopes are shared between the anaphylatoxins and their precursor proteins. For immunohistochemical experiments, we used a human-specific antibody recognizing a neoepitope exposed upon cleavage of C3 allowing specific detection of C3a; however, this antibody did not work in western blots or ELISA. A comparable approach could not be applied for C5 as the corresponding neoepitope-specific antibody against human C5a showed no cross-reactivity in rat samples. In plasma, C3a or its desArg form could be detected by western blots with a rat-specific C3a antibody (also detecting C3 and C3a-desArg) and distinguished from C3 by their molecular size. However, concentrations in the lung were below the detection limit of this method. Furthermore, selective quantification of the desArg forms of C5a and C3a remains technically challenging across species as no antibodies are currently available that distinguish the peptides with and without the C-terminal arginine. Finally, we also cannot exclude the contribution of other substrates of CPM, such as bradykinin and CXCL12,^12^ to the phenotype of CPM-KO rats. Nevertheless, the CVF-induced acute lung injury model is primarily driven by complement activation^14^^,^^15^^,^^16^ and there is currently no evidence implicating bradykinin or CXCL12 in its pathogenesis. Future studies using anti-C3a antibodies in the CPM-KO rats will clarify the mechanism of action.

## Resource availability

### Lead contact

Requests for further information and resources should be directed to and will be fulfilled by the lead contact, Michael Bader (mbader@mdc-berlin.de).

### Materials availability

The rat line generated in this study is available from the lead contact.

### Data and code availability

• Data reported in this study will be shared by the lead contact, upon request.
• This study does not report original code.
• All other relevant data are provided in the article and its supplemental information.

## Acknowledgments

We thank Sabine Meyer from the Protein Production & Characterization Technology Platform at the Max-Delbrück-Center for Molecular Medicine in the Helmholtz Association (MDC), Berlin, Germany (https://www.mdc-berlin.de/ppcp), for technical support during mass spectrometry analyses. We also thank Susanne da Costa Goncalves, Madeleine Skorna-Nussbeck, and Patrick Langner for technical help. This work was supported by research grants from the BMFTR (German Federal Ministry of Research, Technology and Space, grant COMPRare EJP RD JTC-2022).

## Author contributions

F.Q., C.Z., A.S., E.P., K.Z., R.G., B.A.C., and A.F.R. performed the experiments; F.Q., N.A., J.B.P., A.F.R., and M.B. designed study and protocols; F.Q, C.Z., A.S., A.F.R., and M.B. analyzed data, F.Q., A.S., and M.B. wrote the manuscript, and all coauthors revised the manuscript.

## Declaration of interests

All authors declare no competing interests.

## STAR★Methods

### Key resources table

**Table:** 

REAGENT or RESOURCE	SOURCE	IDENTIFIER
**Antibodies**		

anti-rat MPO	Proteintech	Cat. #66177
anti-rat CD68	Proteintech	Cat. #28058
anti-rat C3a	HycultBiotech	Cat. #HM2074
anti-rat C3a	Thermo Fisher	Cat. #PA1-9567
anti-rat CPM	SIGMA-Aldrich	Cat. #HPA002657
IRDye 680RD Donkey anti-Rabbit IgG	LI-COR	Cat. #926-32213
IRDye 800CW Donkey anti-Chicken IgG	LI-COR	Cat. #926-32218
IRDye 680RD Donkey anti-Goat IgG	LI-COR	Cat. #926-32224
anti-rat GAPDH	Cell Signaling	Cat. #2118
anti-rat transferrin	R&D Systems	Cat. #AF3987

**Chemicals, peptides, and recombinant proteins**		

Recombinant CVF	HycultBiotech	Cat. #HC4073
Recombinant C3a	EMD-Millipore	Cat. #204881
Recombinant C5a	Novus Biologicals	Cat. NBP2-61368
Recombinant CPM	R&D Systems	Cat. #7457-ZN
Recombinant CPB1	Roche Diagnostics	Cat. #08039852001
Dansyl-Ala-Arg-OH	Bachem	Cat. #4028360
DL-2-mercaptomethyl-3-guanidinoethylthiopropanoic acid (MERGETPA)	MedChemExpress	Cat. #HY-W704574

**Critical commercial assays**		

M-MLV reverse transcriptase	Promega	Cat. #M1708
GoTaq Probe RT-qPCR System	Promega	Cat. #A6101
RNAScope technology	ACD Bio-Techne	Cat. 322001, 322330, 320058, 322360, 320409
RNAScope probe for rat Cpm mRNA	ACD Bio-Techne	Rn-Cpm-C1, Lot#21211B
Eukitt	SIGMA-Aldrich	Cat. #03989
Fluoromount-G	Invitrogen	Cat. #00-4959-2

**Experimental models: Organisms/strains**		

CPM-KO rat	This study	N/A

**Oligonucleotides**		

See Table S1 for oligonucleotides information	N/A	N/A

**Software and algorithms**		

G Power 3.1	Heinrich-Heine University Düsseldorf, Germany	https://www.psychologie.hhu.de/arbeitsgruppen/allgemeine-psychologie-und-arbeitspsychologie/gpower
Prism Version 6.04	GraphPad	https://www.graphpad.com

### Experimental model and study participant details

#### Generation of CPM-Knockout rats by CRISPR/Cas9 mediated genome editing

State of Berlin authorities (LAGESO) approved the generation of the CPM-Knockout (KO) rats (license No. G0162/14) confirming that all experiments conform to the relevant regulatory standards. Animals were housed in a temperature-controlled (21 ± 1 °C) and humidity-controlled (40–50%) colony room under a 12/12 h light/dark cycle (lights on at 06:00 h). The rats were group-housed (4 rats/cage) under specific-pathogen free conditions with free access to food and water. The rat model was generated by electroporation of Sprague-Dawley (SD, JANVIER Labs, France) rat two-cell embryos with a mixture of 1280 ng/μL Cas9 protein (IDT), 258 ng/μL sgRNA with the sequence 5′-UUCCAUCUCUAGGACAUGGACGG (IDT) to target the region in the rat *Cpm* gene covering the ATG start codon, and 500 ng/μL tracrRNA (Dharmacon). The electroporated embryos were transferred into foster mothers according to established methods.^30^ The offspring were genotyped by polymerase chain reaction with primers flanking the gRNA target region (CPM5, 5′-GTGGGACCATCTTTACCTGG; CPM3, 5′-TGTAGTTCTGAGCCACGCTC) and sequencing of the polymerase chain reaction fragment.

#### Cobra venom factor (CVF)–induced acute lung injury

State of Berlin authorities (LAGESO) approved induction of acute lung injury in rats (license No. G0001/23) confirming that all experiments conform to the relevant regulatory standards. Complement-mediated acute lung injury was induced in 12–15-week-old male wildtype SD and CPM-KO rats by intravenous administration of CVF. Anesthesia was achieved using isoflurane for induction (3–5%) and maintenance (1–2%) mixed with room air, delivered via a small-animal anesthesia unit (Univentor #U-410, AgnThos). Following induction of anesthesia, rats were implanted with a saline-filled catheter into the inferior vena cava via the femoral vein. The catheter was used for blood sampling and intravenous CVF administration. Recombinant CVF (HycultBiotech #HC4073) derived from *Naja kaouthia* was administered as an intravenous bolus at a dose of 4IU/kg, equivalent to 14 μg/kg. Control rats received a sham injection of 0.9% NaCl instead of CVF. For analgesia, rats received an intraperitoneal injection of carprofen (5 mg/kg) prior to surgery. Approximately 100 μL of blood was collected before CVF administration and at 10, 20, 30, and 60 min post-administration to quantify the changes in the circulating immune cells. Blood samples were collected into EDTA-coated tubes, centrifuged at 4000xg at 4°C for 5 min. One hour after CVF administration, rats were euthanized by increasing the isoflurane concentration to 5%, followed by exsanguination, during which additional EDTA plasma and serum samples were obtained and lung tissue was harvested for histological and gene expression analyses.

Blood samples were kept at room temperature until cell counting. Hematological analyses were performed using an automated hematology analyzer (IDEXX ProCyte DX) at the animal phenotyping facility of the Max Delbrück Center for Molecular Medicine, Berlin.

The influence of sex on the results of the study cannot be excluded and the use of only male rats is therefore a limitation to the results’ generalizability.

### Method details

#### CPM enzyme activity assay

CPM enzyme activity was quantified with a modified fluorometric assay^31^ with slight modifications. Lung tissue was homogenized in ice-cold PBS, centrifuged and total protein concentration was measured using the BCA method and aliquoted in 50 μg protein/10 μL. In brief, the reaction was carried out in 250 μL of 0.1 M HEPES, pH 7.5 containing 50 μg protein from lung homogenate and 100 μM of the substrate Dansyl-Ala-Arg-OH at 37°C for 3 h. Specificity of the assay was verified by adding DL-2-mercaptomethyl-3-guanidinoethylthiopropanoic acid (MERGETPA) (10 μM). Reactions were stopped by adding 150 μL of 1M sodium citrate (pH 3.1), extracted with 1 mL of chloroform, vortexed, and centrifuged (2500 rpm for 10 min). Dansyl-Ala in the chloroform phase was measured spectrofluorometrically (340 nm excitation, 495 nm emission).

#### Carboxypeptidase metabolism of C3a and C5a assessed by intact mass spectrometry

*In vitro* carboxypeptidase assays were performed to assess whether human recombinant CPM (cat. #7457-ZN, R&D) and CPB1 (Ref. #08039852001, Roche Diag. Mannheim, Germany) catalyze the release of the C-terminal arginine from complement components C3a (cat. #204881, EMD-Millipore, affiliated Merck, Germany) or C5a (NBP2-61368, Novus Biologicals, Germany). Protein substrates were used at 4 μg in 20 μL reaction buffer (0.2 M HEPES, pH 7.0). Control reactions contained C3a or C5a protein only, whereas test reactions additionally included 100 ng recombinant CPM or CPB1. Reactions were incubated for 5 min at 37°C and subsequently stopped by addition of 2.5 mM EDTA prior to analysis

For intact mass analysis, samples were denatured and reduced in 5 M urea and 40 mM DTT for 10 min at room temperature. Intact mass measurements were performed based on the workflow described by Chalk^32^ with the following specifications. Five to ten microliters were injected onto an Agilent 1290 Infinity II UHPLC coupled to an Agilent 6230B TOF mass spectrometer equipped with an Agilent Jet Stream (AJS) ion source operated in positive mode. Online desalting was performed on a Zorbax 300SB-C3 guard column (2.1 × 12.5 mm, 5 μm; Agilent) with 0.1% (v/v) formic acid in water (A) and methanol (B) as mobile phases. Source parameters were: capillary voltage 4000 V, nebulizer pressure 50 psi, drying gas 350 °C at 12 L/min, and sheath gas 350 °C at 11 L/min; ion optic voltages were 250 V (fragmentor), 65 V (skimmer), and 750 V (octupole RF). Spectra were deconvoluted using the Protein Deconvolution module in MassHunter BioConfirm v10.0 (Agilent Technologies) applying the Maximum Entropy algorithm over an m/z range of 800–2,500 with a signal-to-noise threshold >30:1.

#### RT-qPCR analyses of gene expression

The tissue was transferred to tubes containing ceramic beads and 1 mL of TRIzol (Invitrogen) and homogenized using a FastPrep 24 homogenization device (MPI). The amount of total RNA was measured using a NanoDrop 1000 (Thermo Fisher). cDNA was prepared by incubating 1 μg of RNA with random hexamers and M-MLV reverse transcriptase (Promega) according to the manufacturer’s instructions. cDNA was diluted to a final concentration of 1 ng/μL. RT-qPCR was performed using SYBR green reagent mix (Promega) following manufacturer’s instructions. The primers for gene amplification (Table S1) were synthesized by Eurofins Genomics (Ebersberg, Germany). A QuantStudio 5 device (Thermo Fisher) was used for RT-qPCR cycling. The Ct values obtained in the exponential phase of amplification from the target gene and the housekeeping genes 18SrRNA and TBP (TATA-binding protein) were taken to calculate the gene expression relative to the sham group. The Livak and Schmittgen method (2^-ΔΔCt^) was used to calculate the relative gene expression.^33^

#### Histological analysis

Lung tissues were fixed in 4% formalin for 24 h. The lung samples were dehydrated in increasing concentrations of ethanol and subsequently embedded in paraffin, 5 μm thick sections were mounted onto slides and kept overnight at room temperature. Sections were deparaffinized in xylene, rehydrated in decreasing concentrations of ethanol and kept in distilled water until further staining.

a) *In situ* hybridization: The tissue localization of CPM mRNA was performed using RNAScope technology (ACD Bio-Techne, Newark, USA) according to the manufacturer’s protocol. Following the deparaffinization and rehydration the endogenous peroxidase was blocked by incubating the sections with H_2_O_2_ at room temperature. The sections were then heated in antigen retrieval buffer (322001, ACD) and digested by proteinase solution at 40°C for 30 min (RNAScope H_2_O_2_ & Protease Plus Reagents, 322330, ACD). Sections were incubated with ISH target probe pairs at 40°C in a hybridization oven for 2 h. The 20 ZZ-ISH probes targeting rat *Cpm* mRNA were designed and synthesized by Advanced Cell Diagnostics (ACD) (Rn-Cpm-C1, Lot#21211B). The sections were washed with washing buffer (320058, ACD). The signal was amplified using the pre-amplifier and amplifier conjugated to alkaline phosphatase and incubated with a Fast Red substrate solution for 10 min at room temperature (RNAScope 2.5 HD Detection Reagent-RED, 322360, ACD). Sections were then counter stained with hematoxylin for 1–2 min, incubated in xylene, and coverslipped using xylene based mounting medium (320409, ACD). Next day images were taken using the inverse bright light/fluorescence Microscope (Keyence BZ-X810, Germany) for evaluation with Keyence BZ-X800 Analyzer software.

b) Sections were stained with H&E according to standard methods, coverslipped with Eukitt (03989, SIGMA-Aldrich), and kept overnight at room temperature. The sections were observed under an inverted light/fluorescence microscope (Keyence BZ-X810, Tokyo, Japan). Lung tissue damage was evaluated by a scoring system described by Matute-Bello et al.^19^

c) For immunofluorescence staining the sections were deparaffinized and rehydrated and boiled in antigen retrieval (AR) buffer either in Tris/EDTA pH 9.0, Citrate buffer pH 6.0 for 10–20 min. Nonspecific bindings were blocked using a mixture of 10% normal donkey serum and 2% bovine serum albumin at room temperature for 30 min. For the staining of complement factors, the sections after AR were heated in antigen-retrieval buffer (322001, ACD) and then treated with ProteasePlus (322330, ACD) for 30 min. at 40°C in an incubator. Subsequently, the sections were incubated with following antibodies; anti-MPO (1:500, Cat. #66177, Proteintech), anti-CD68 (1:500, Cat. #28058, Proteintech), anti-C3a (1:1000, Cat. #HM2074, Hycult Biotech), overnight at 4°C. Next day the sections were washed and incubated with fluorescein-conjugated secondary antibodies in a concentration of 1:300 for 2 h at RT, washed and cover-slipped with Fluoromount-G with DAPI mounting medium (00-4959-2, Invitrogen). The sections were examined with an inverted bright light/fluorescence microscope (Keyence BZ-X810).

#### Western Blot

Lung tissue was homogenized in RIPA buffer (Abcam) supplemented with protease inhibitor cocktail (complete, Roche), and EDTA-plasma was diluted in distilled water (1:10, v/v). The extracted proteins were resolved on a 10% and 15% SDS-PAGE gel and subsequently transferred onto nitrocellulose membranes (Thermo Scientific, USA) using a Bio-Rad wet transfer system (Hercules, CA, USA) and tank transfer. Membranes were incubated with primary antibodies against rat CPM (1:1000; Sigma cat. #HPA002657) or C3a (1:1000, Thermo Fisher, #PA1-9567) overnight at 4°C. Membranes were washed in PBS-T (PBS containing 0.2% Tween 20) and incubated with secondary antibodies (1:10,000; LI-COR #926–32213; LI-COR #926–32218) for 2 h at room temperature. Protein levels were normalized to GAPDH (1:1000; Cell Signaling, #2118) or transferrin (1:1000, R&D System, #No.AF3987) and secondary antibody (1:10,000 LI-COR, #926–32224) following the same protocol as above. Detection was performed with an Odyssey DLx Imaging System (LI-COR Biosciences, Lincoln, NE, USA) using chemiluminescence. Quantitative analysis of the protein bands was carried out with ImageJ software.

### Quantification and statistical analysis

Power analysis was applied using the software G Power 3.1. As parameters for power analysis, type I error was assumed at a significance level of 0.05 and power of 80%. Data are presented as mean ± SD, including scattered plots in bar graphs. Statistical analyses were performed using GraphPad Prism. Sample sizes were determined based on power calculations (α = 0.05, power = 0.8) to detect biologically relevant differences. Differences between groups were assessed by two-way ANOVA (factors: [Genotype] and [Treatment]), followed by Tukey’s multiple comparisons test when appropriate. Also, unpaired two-tailed Student’s *t* test was used as indicated in the figures, where appropriate. Across all analyses statistical significance was defined as *p* < 0.05.
