# Supplementary material for: Carboxypeptidase M deficiency aggravates complement-induced lung injury
Source: iScience. 2026 Sep 5;29(9):117463. doi: 10.1016/j.isci.2026.117463 (PMC13594428; doi:10.1016/j.isci.2026.117463)
Supplement: Supplementary file 1 [file mmc1.pdf]

iScience, Volume *29*

Supplemental information

Carboxypeptidase M deficiency aggravates complement-induced lung injury

Fatimunnisa Qadri, Chubin Zhang, Anja Schütz, Elena Popova, Beatriz Atocha Czaplinska, Karina Zemechmann, Ramin Greckl, Natalia Alenina, Joao B. Pesquero, André F. Rodrigues, and Michael Bader

Supplemental information

Figure S1: Carboxypeptidase enzyme activity in kidney and bone marrow and CPN1 and CPB2 expression in lung and liver

Carboxypeptidase activity is strongly reduced in the kidney (A) and bone marrow (B) of CPM-KO rats. The expression of CPN1 is unchanged in lung (C) and liver (E) and CPB2 expression is unchanged in lung (D) but downregulated in liver (F) of CPM-KO rats. Cobra venome factor (CVF) treatment does not significantly change CPN1 and CPB2 expression in lung. SD, Sprague Dawley control rats, CPM-KO, CPM-knockout rat, MERGETPA, DL-2-mercaptomethyl-3-guanidinoethylthiopropanoic acid. n=4/group ****, p<0.0001

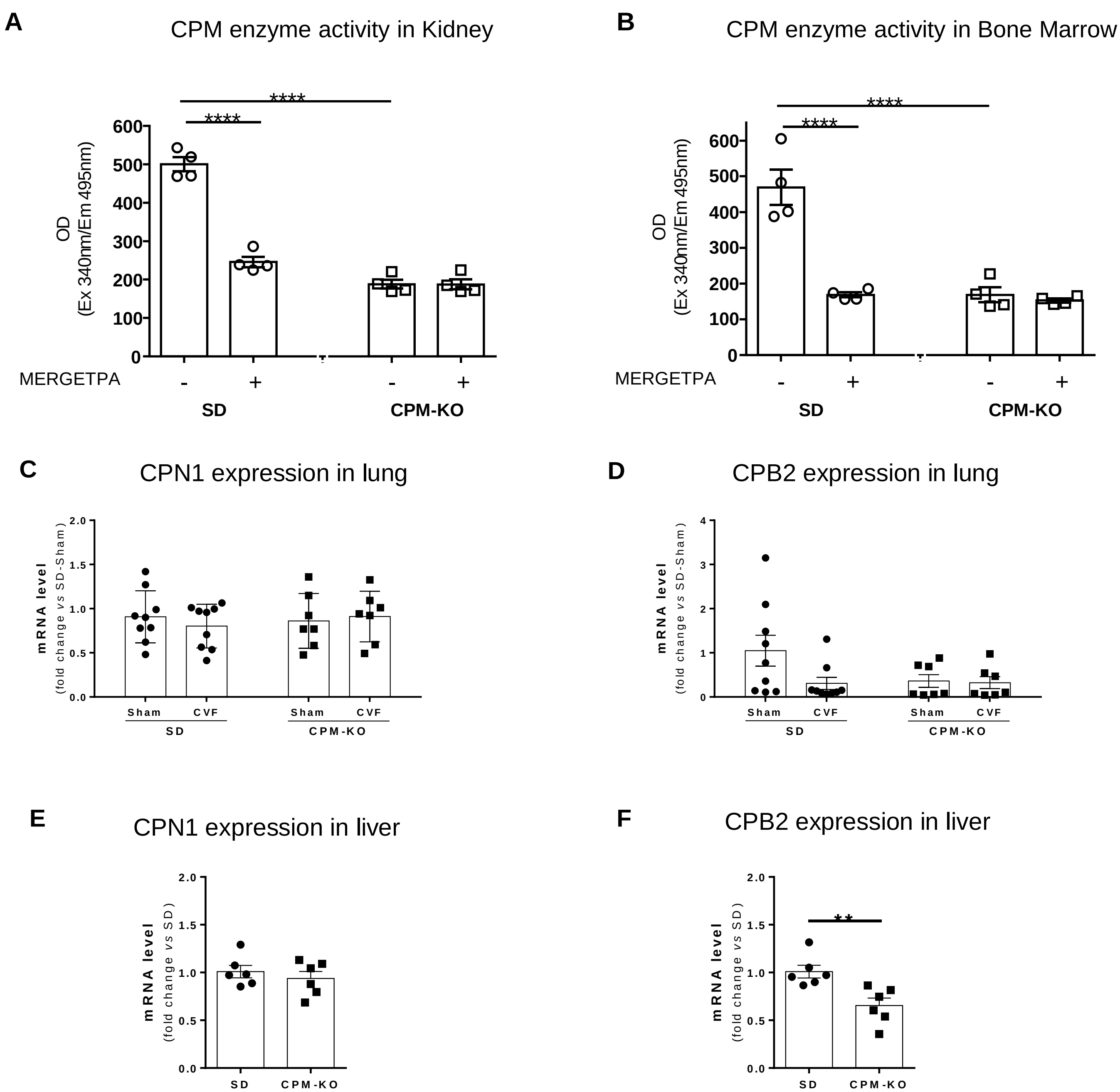

Table S1. Oligonucleotides

All oligonucleotides were synthesized by Eurofins Genomics (Ebersberg, Germany)

CPM5 5′-GTGGGACCATCTTTACCTGG-3’;

CPM3 5′-TGTAGTTCTGAGCCACGCTC-3’

MPO sense 5´- CTACCCGGTTCTCCTTCCTC -3´

MPO antisense 5´- GGTTCTTGATTCGAGGGTCA -3´,

CD11b (Itamg) sense 5´- GGCTCCGGTAGCATCAACAG-3´

CD11b (Itamg) antisense 5´- ATTTTGGGTCAGGGTTTCTCT -3´,

Itgb2 sense 5´- GAGGAATGCACCAAGTACAAAGT-3´

Itgb2 antisense 5´- GTCACAGCGCAAGGAGTCA-3´,

MCP-1 (Ccl2) sense 5´- GATGCAGTTAATGCCCCACT-3´

MCP-1 (Ccl2) antisense 5´- ACCTGCTGCTGGTGATTCTC-3´,

C5aR1 sense 5´-GTCAACGCCATTTGGTTTCT-3´

C5aR1 antisense 5´-GGGCCAGTGGTTATGCTTTA-3´,

TGF-ß sense 5´-GCAGAGTTCAGGGTCTTTCG-3´

TGF-ß antisense 5´-GCTGGGTTGGAGATGTTAGG-3´,

TNF-α sense 5´-ATGGGCTCCCTCTCATCAGTTC-3´

TNF-α antisense 5´-GCTTGGTGGTTTGCTACGAC-3´,

IL-6 sense 5´-GCCCTTCAGGAACAGCTATG-3´

IL-6 antisense 5´-TGAAGTAGGGAAGGCAGTGG-3´,

IL-1ß sense 5´-CAGCTTTCGACAGTGAGGAGA-3´

IL-1ß antisense 5´-TTGTCGAGATGCTGCTGT GA-3´,

CD68 sense 5´-ACCAATCTCTCTTGCTGCCT-3´

CD68 antisense 5´-GAGGGGCTGGTAGGTTGAT-3´,

CPB2 sense 5´-GTGTGAGAAAGGCGCATCAA-3´,

CPB2 antisense 5´-TTCTCCTCAGGAAGTCAGCC-3´,

CPN1 sense 5´-GGTTCGGGTCACTGCATACT-3´,

CPN1 antisense 5´-GACAACTTCTTGCAGCACCA-3´,

18S sense 5´-CGTTCTTAGTTGGTGGAGCG3´

18S antisense 5´-AACGCCACTTGTCCC TCTAA-3´

TBP sense 5´-ACTGTTTCATGGTGCGTGAC-3´

TBP antisense 5´-AGCATAAGGTGGAAGGCTGT-3´
